# Delayed posthypoxic leukoencephalopathy following alcohol and psychotropic drug overdose: a case report

**DOI:** 10.1002/ccr3.1544

**Published:** 2018-05-02

**Authors:** Kanji Ueno, Tetsuya Takahashi, Masato Higashima, Ryoko Okazaki, Seiichiro Takano, Yuji Wada

**Affiliations:** ^1^ Department of Neuropsychiatry Faculty of Medical Sciences University of Fukui Fukui Japan; ^2^ Health Administration Center University of Fukui Fukui Japan; ^3^ Department of Neurology Fukui Red Cross Hospital Fukui Japan

**Keywords:** Delayed posthypoxic leukoencephalopathy, hypoxia, magnetic resonance imaging, supplementary biomarker, vascular endothelial growth factor

## Abstract

Delayed posthypoxic leukoencephalopathy (DPHL), a demyelinating syndrome, can easily be misdiagnosed as a psychiatric condition. Our case study shows that magnetic resonance imaging is highly useful for an early diagnosis of DPHL and that vascular endothelial growth factor might be a supplementary biomarker for the early detection of DPHL.

## Introduction

Delayed posthypoxic leukoencephalopathy (DPHL) is a rare type of demyelinating syndrome that can be caused by any insult leading to a prolonged period of cerebral hypo‐oxygenation 1. DPHL is clinically characterized by a variety of neuropsychiatric symptoms and by a lucid interval after the initial recovery from the prolonged hypoxic insult to the brain 2. Due to its complex clinical features and lucid interval, DPHL can be mistaken as the onset of another neurological or psychiatric condition. Furthermore, although many patients with DPHL demonstrate spontaneous remissions, even if incomplete, some patients show poor clinical outcomes. Therefore, an early diagnosis of DPHL is important to avoid unnecessary treatments or investigations 1. However, the exact pathogenic mechanisms of DPHL remain elusive, and a well‐defined therapeutic strategy has not been established.

Vascular endothelial growth factor (VEGF), a key determinant of vascular permeability, plays a major role in neurogenesis, angiogenesis, and functional recovery after hypoxia 3. Given these known roles, we hypothesized that VEGF expression served as a predictive biomarker of DPHL.

This report presents a case of DPHL due to hypoxia, following an overdose of alcohol and psychotropic drugs. Herein, we describe the changes in the serum levels of VEGF, alongside magnetic resonance imaging (MRI) findings, and their relationship with the development of clinical symptoms.

## Case Presentation

A 63‐year‐old nonsmoking Asian man with a 12‐year clinical history of bipolar I disorder had attempted suicide by overdosing on psychotropic agents after the consumption of a large amount of alcohol. His wife found him unconscious while holding a kitchen knife in his right hand in a bedroom. She also found an empty bottle of whiskey and empty blister packs from several drugs (zolpidem, etizolam, lamotrigine, and lithium carbonate). The exact time that had elapsed between the overdose and her discovery was unknown, and his wife called emergency medical assistance.

**Figure 1:**
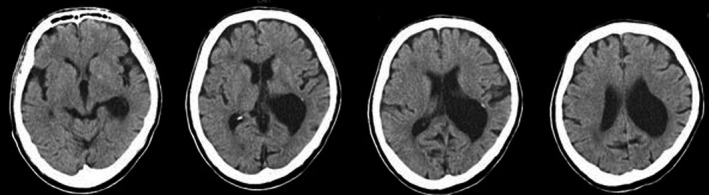
Brain computed tomography (CT) scans immediately after the hypoxic insult.

On arrival at the emergency room, the patient was in a deep coma with hypotension (systolic blood pressure: 50 mmHg), tachycardia (heart rate: 90 beats/min), and tachypnea (30 breaths/min). Arterial blood gas sample analysis revealed respiratory acidosis and hypoxemia (pH: 7.14, PaCO_2_: 48.1 mmHg, PaO_2_: 81.1 mmHg, HCO_3_: 15.7 mmol/L, adjusted base excess: −13.2 and SaO_2_: 87.7%). Blood examination revealed liver dysfunction (aspartate aminotransferase (AST): 1107 IU/L; alanine aminotransferase (ALT): 344 IU/L), renal dysfunction (creatinine: 2.75 mg/dL), and leukocytosis (white blood cell count: 16.2/mL), with increased creatine phosphokinase (CPK, 65452 IU/L) and blood urea nitrogen (26.1 mg/dL). Blood sugar was 176 mg/dL. An examination with Triage DOA^®^ (Sysmex Corp., Japan), which is a simple drug screening kit that utilizes urine samples, was difficult due to a decrease in the urinary volume. Computed tomography (CT) scanning of the head was negative except for a paraventricular leptomeningeal cyst (Fig. 1).

The patient was intubated, mechanically ventilated, and moved to an intensive care unit. He was successfully resuscitated using isotonic sodium chloride solution, bicarbonate Ringer's solution, and noradrenaline. Two days later, he recovered consciousness and could converse with the medical staff. He gradually demonstrated favorable physical recovery. However, he underwent dialysis because of residual acute renal dysfunction (creatinine: 4.65 mg/dL), liver dysfunction (AST: 808 IU/L and ALT: 270 IU/L), and an increased CPK level (33,028 IU/L). After 10 days of intensive care, he moved to a general ward. Because of his suicidal ideation, he was examined by a psychiatrist. On examination, he was completely lucid, and he was able to conduct his daily activities entirely independently. However, he reported depressive symptoms, including depressed mood, anxiety, feelings of guilt, and suicidal ideation. On the 24th day of admission, after good physical recovery that was not accompanied by cognitive deficits, he was transferred to the psychiatric ward of our hospital due to his residual depressive symptoms.

**Figure 2:**
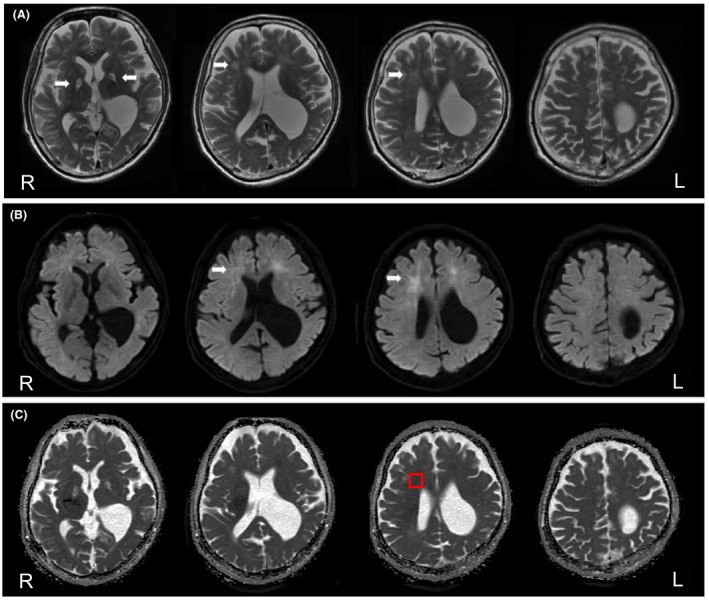
Magnetic resonance imaging (MRI) of the brain 26 days after the hypoxic insult. (A) T2 images reveal confluent hyperintense regions in the bilateral frontal white matter and the globus pallidus (arrows). (B) Diffusion‐weighted imaging (DWI) reveals hyperintensity regions in the white matter corresponding to the changes seen in the T2 images (arrows). (C) An apparent diffusion coefficient (ADC) map reveals no obvious hypointensity regions in the white matter corresponding to the changes seen in the T2 images and DWI.

On admission, the patient was taciturn and dull. We considered these as partial symptoms of depression, and the patient was initiated on aripiprazole. After 2 days in our hospital (day 26 from initial admission), he appeared abruptly confused and demonstrated disorganized behavior, such as urinating into a washbowl, and the disorganized behavior rapidly worsened over the following days. We immediately stopped the administration of aripiprazole. A neurological evaluation revealed an impaired orientation, an inability to follow instructions, delayed responses, and urinary incontinence. Urinalysis, electrocardiogram, and chest X‐ray revealed no remarkable changes. The results of a blood count, serum chemistry analyses, and ammonia, vitamin B12, and thyrotropin measurements were normal. Nonetheless, the patient had persistent anemia (red blood cell: 2.53 × 10^6^/μL, Hb: 7.7 g/dL) and hypokalemia (2.5 mEq/L) due to irreversible renal failure. Electroencephalography (EEG) revealed continuous diffuse polymorphic 3–10‐Hz delta‐alpha waves, without paroxysmal discharge. T2‐weighted brain MRI revealed confluent hyperintense regions in the bilateral frontal white matter and the globus pallidus (Fig. 2A, top panels). This hyperintensity in the white matter was also noted on diffusion‐weighted imaging (DWI) (Fig. 2B, middle panels). On the other hand, the apparent diffusion coefficient (ADC) map did not show appreciable changes in the corresponding frontal white matter except for rather hyperintense regions in the globus pallidus (Fig. 2C, bottom panels). In many previously reported cases 4, 5, 6, 7, 8, 9, 10, 11, 12, 13, changes that were observed in the more rostral regions were more dramatic than those in the periventricular region. However, in this case, the changes in the periventricular region were the most dramatic, and there were few changes in the more rostral regions, such as the centrum semiovale (Fig. 2A–C, rightmost). Magnetic resonance spectroscopy (MRS) of the white matter showed decreased N‐acetylaspartate to total creatinine ratios, increased total choline to creatinine ratios, and increased lactate to creatinine ratios, suggestive of neuronal damage. Within a few days, the patient developed akinetic mutism. Subsequently, he showed apparent frontal lobe syndrome, with symptoms such as a grasping reflex, pathological laughing, irritability, apathy, perseveration, and stereotyped behaviors.

**Figure 3:**
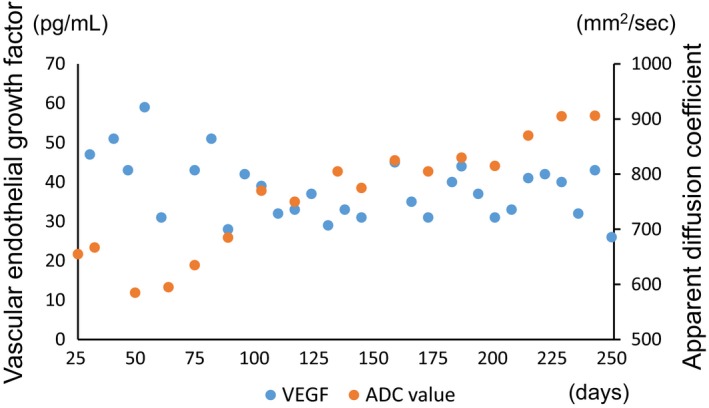
Changes in the plasma concentration of vascular endothelial growth factor (VEGF) between 31 days after the hypoxic insult and the seven‐month follow‐up. Additionally, changes in the ADC between 26 days after the hypoxic insult and the seven‐month follow‐up are shown.

Given the patient's clinical course and neuroimaging findings, we diagnosed him with DPHL due to hypoxia, secondary to alcohol‐ and benzodiazepine‐induced respiratory depression. Therefore, we examined arylsulfatase A, an enzyme that is essential for the turnover of myelination, which reportedly indicates DPHL 14, 15. The patient exhibited normal levels of arylsulfatase A, which was consistent with recent case reports 16, 17. We additionally examined the plasma concentrations of VEGF because the expression of this protein increases in patients after stroke 18. Interestingly, VEGF was elevated to 47 pg/mL (reference value: <38.5 pg/mL) 7 days after admission to our hospital, and except at two points, this elevation lasted approximately 100 days after the hypoxic insult (Fig. 3). Furthermore, we set the region of interest (ROI) in the corresponding frontal white matter region, as shown in Figure 2 (a section surrounded by a red rectangle), and calculated the average ADC of the ROI. The ADC showed relatively low values for approximately 100 days compared to subsequent days (Fig. 3).

**Figure 4:**
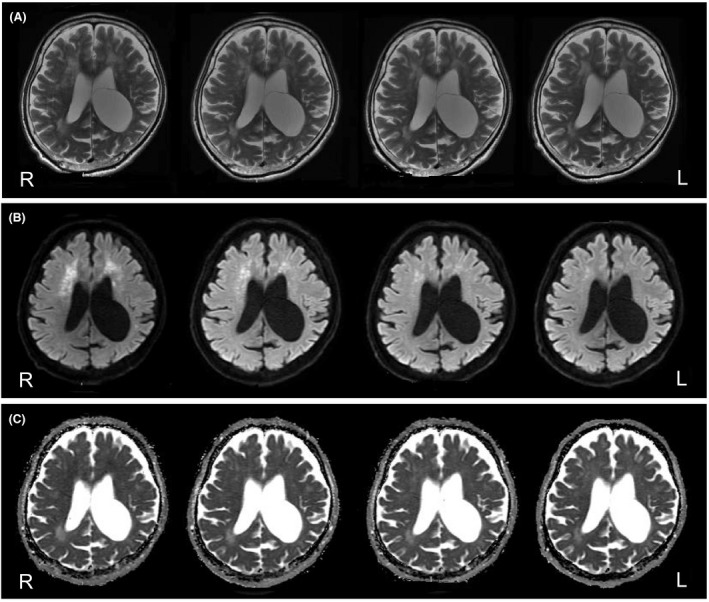
Follow‐up MRIs obtained every 2 months (from left to right). Left periphery images were obtained approximately 2 months after the hypoxic insult. (A) T2 images reveal slight deterioration of the periventricular regions. (B) DWI reveals hyperintensity in the corresponding periventricular regions and that the high signals gradually weakened. (C) The ADC maps reveal slight changes in the corresponding periventricular regions.

**Figure 5:**
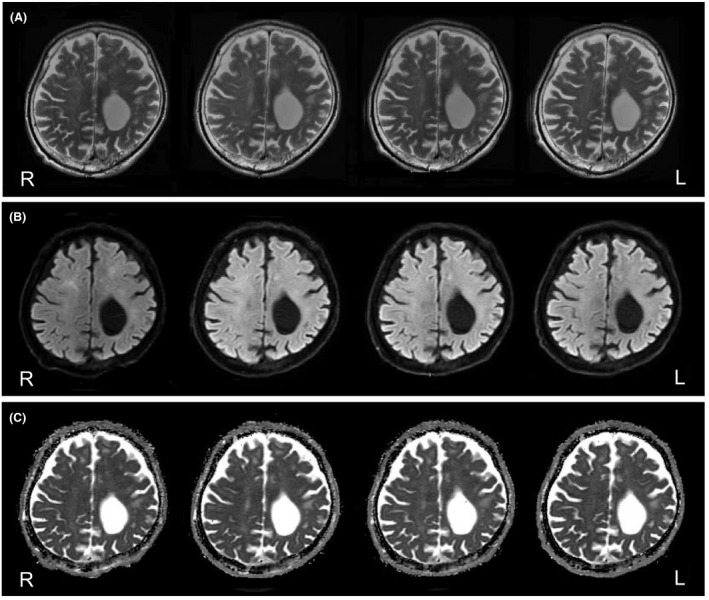
Follow‐up MRIs obtained every 2 months (from left to right). Left periphery images were obtained approximately 2 months after the hypoxic insult. (A) T2 images reveal no remarkable changes in the centrum semiovale regions. (B) DWI reveals no remarkable changes in the centrum semiovale regions. (C) The ADC maps reveal no remarkable changes in the corresponding centrum regions.

Seven weeks after the onset of symptoms, he demonstrated gradual improvements in responsiveness, alertness, and the activities of daily living. He had responded well to valproic acid for impulsivity and partially responded to memantine for apathy, whereas donepezil hydrochloride was not effective. Thereafter, he was no longer mute, and he could follow instructions. However, significant symptoms of frontal lobe damage (i.e., apathy, disorientation, perseveration, stereotypy, and impulsivity) remained. During hospitalization, we repeatedly examined the patient using EEGs, MRIs, and blood examinations, including measurements of VEGF levels. Alterations observable on EEG examination did not change throughout the hospitalization period. Although hyperintensity on T2 MRI (Fig. 4A, top panels) and MRS revealed slight deteriorations of the periventricular regions, the hyperintensity of the corresponding regions on DWI (Fig. 4B, middle panels) gradually weakened, and the hyperintensity of the globus pallidus on T2 MRI was completely improved. Furthermore, the ADC map (Fig. 4C, bottom panels) showed slight changes in the corresponding periventricular regions. Indeed, the ADC values indicated transitions, as shown in Figure 3. In contrast, during hospitalization, there were few changes in the centrum semiovale regions on MRI (Fig. 5A–C). On the other hand, the VEGF levels tended to fluctuate around the reference value after 100 days (Fig. 3).

At the follow‐up visit after 6 months of hospitalization, despite persistent frontal symptoms, the patient was performing at his previous neurological baseline. In the end, he was transferred to a chronic care hospital where he continued with a regimen of valproic acid 600 mg/day and memantine 20 mg/day.

## Discussion

This case report describes DPHL after a patient with bipolar disorder overdosed on psychotropic drugs after consuming a large amount of alcohol. Notably, drug overdoses are not rare in patients with mental disorders. Psychiatrists frequently encounter such situations in daily clinical consultations. Regarding the clinical course, our patient's taciturnity and loss of activity following a lucid interval was likely an initial sign of DPHL. However, considering that the patient was in a depressive state at the time, some signs of DPHL certainly seemed to be consistent with depressive symptoms. The exact diagnosis was promptly confirmed following the abrupt onset of a clear frontal lobe syndrome and the observation of some apparent abnormalities on the MRI scan. Recently, Zamora et al. 4 reviewed DPHL cases due to respiratory failure in drug and alcohol overdoses and their relationships with MRI findings. The authors claim that specific MRI findings are highly suggestive of DPHL, in the appropriate clinical setting. From this case, we learned that DPHL can easily be misdiagnosed, especially when a patient is suffering from comorbid mental disorders. Therefore, when DPHL is suspected, MRI is crucial for determining the diagnosis.

Despite relatively minor changes on MRI, our patient experienced a poor outcome, as observed for many previous patients who have overdosed of benzodiazepines and alcohol 4, 5, 6, 7, 8, 9, 10. The clinical presentation of our patient was complicated by mild chronic anemia (Hb 10–12 g/dL), although he had no history of anemia. The low oxygen‐carrying capacity of the blood due to anemia leads to anemic anoxia. Indeed, a decreased hemoglobin content may cause anemic hypoxia 19. Furthermore, Lou et al. 20 reported a case of delayed postanoxic leukoencephalopathy after hypoxic–ischemic injury. Their patient had a history of anemia caused by untreated chronic blood loss. The authors speculated that previous anemia might have played a role in subsequent hypoxic–ischemic brain injury. Consequently, we might assume that a poor clinical outcome that was not indicated by the MRI findings, as seen in our case, may be due to the coexistence of prolonged anemia even after the lucid interval. If this speculation is correct, the implications for treatment may be important because controlling anemia would potentially lead to a better clinical outcome.

Another important consideration is that this report is the first to describe the VEGF level in a patient with DPHL. The plasma concentration of VEGF was increased for approximately 100 days after the hypoxic insult. Cerebral white matter is supplied primarily by widely spaced arterioles with few anastomoses and thus may be less able than gray matter or the posterior fossa to compensate for hypoxia or hypoperfusion 21. Therefore, oligodendrocytes, which are the myelin‐producing cells of the central nervous system and require higher levels of oxygen and glucose than other glial types, might be particularly vulnerable to hypoxia due to their relative lack of collateral blood flow 1, 2. On the other hand, VEGF expression reportedly promotes neovascularization and the formation of microvascular networks in hypoxic–ischemic brain encephalopathy 22. Human clinical studies involving measurements of VEGF levels following stroke have reported that serum VEGF increases immediately after the onset of stroke in human patients 18 and that the increase continues for at least 90 days for all stroke types 23. Furthermore, the administration of erythropoietin in anoxic rats has been shown to enhance angiogenesis, reduce white matter damage, and promote cognitive recovery through the VEGF/VEGF receptor signaling pathway 24. Interestingly, the early administration of erythropoietin to patients with carbon monoxide poisoning improves neurological outcomes and reduces the incidence of delayed neurological sequelae 25.

Our patient demonstrated relatively elevated VEGF levels, even after the lucid interval. This elevated level may have been much higher immediately after the hypoxic insult. Interestingly, as shown in Figure 3, the levels of VEGF showed an upward trend, and conversely, the ADC showed a downward trend. In addition, both trends lasted approximately 100 days, and these results were consistent with those of a previous report 23. We therefore speculate that the VEGF level after a hypoxic insult might be a supplementary biomarker for the early detection of DPHL, although more cases are needed to ensure the validity of our speculation.

## Authorship

KU: was involved in the direct care of the patient, performed the psychiatric evaluation, acquired the clinical data, managed the literature search, and wrote the first draft of the manuscript. TT: supervised the care of the patient, managed the literature search, and critically revised the manuscript. MH: performed the psychiatric evaluation and neuroradiological examination. RO: helped with the collection of clinical data and with the analysis of EEG data. ST and YW: provided critical comments and revised the manuscript.

## Conflict of Interest

The authors declare that they have no competing interests.

## Consent for Publication

Written informed consent was obtained from the relative of the patient (the patient's legal guardian) for the publication of this case report and any accompanying images. A copy of the written consent form is available for review by the Editor‐in‐Chief of this journal.

## Availability of Data and Materials

The dataset supporting the conclusion of this article is included within the article. All clinical data are stored in an information system at the Department of Neurology, Fukui Red Cross Hospital and Department of Neuropsychiatry, Faculty of Medical Sciences, University of Fukui, Japan.
